# Assisted Da Vinci robotic surgery combined with 3D printing technology applied in septal myectomy

**DOI:** 10.1186/s40001-023-00985-z

**Published:** 2023-01-10

**Authors:** Shuaipeng Zhang, Zhuang Liu, Haiyuan Liu, Chengxin Zhang

**Affiliations:** Department of Cardiovascular Surgery, First Affiliated Hospital of Anhui Medical University, Hefei, 230032 China

**Keywords:** 3D printing technology, Da Vinci robotic surgery, Hypertrophic obstructive cardiomyopathy, Minimally invasive, Septal myotomy

## Abstract

Generally, the standard surgical route of Morrow begins with the incision of median sternal, which leads to more trauma, pains and discomforts to patients with hypertrophic obstructive cardiomyopathy (HOCM). It is more difficult and rough to perform the competed resection of hypertrophic myocardium due to complicated anatomical morphology of left ventricular outflow tract and limited visual field of left ventricle during surgery. As the novel surgical strategy, firstly, under the guiding of 3D printing technology, the platform of effective preoperative evaluation focusing on how to resect the hypertrophic myocardium is established. Then, combined with assisted Da Vinci robotic surgery system, the outcome of patient with HOCM is positive and promised.

## Introduction

It has been demonstrated that HOCM is a kind of the autosomal dominant inherited diseases, characterized mainly as left ventricular hypertrophy, especially for ventricular septum [1]. After the development of surgical facilities and surgeons’ experience based on the practical and theoretical updates on pathophysiology of HOCM, clinically, both modified surgical conception and strategy concentrating on minimal invasive procedure, less traumatic injury and faster postoperative recovery are emphasized and considered as the predominate and optimal surgical mode in future. In our case report focusing on the surgical treatment of HOCM, assisted Da Vinci robotic surgery combined with 3D printing technique are applied in the surgical treatment of septal myectomy. The details for both procedure and outcome are presented as follows.

## Case presentation

### Preoperative preparation

**Fig. 1:**
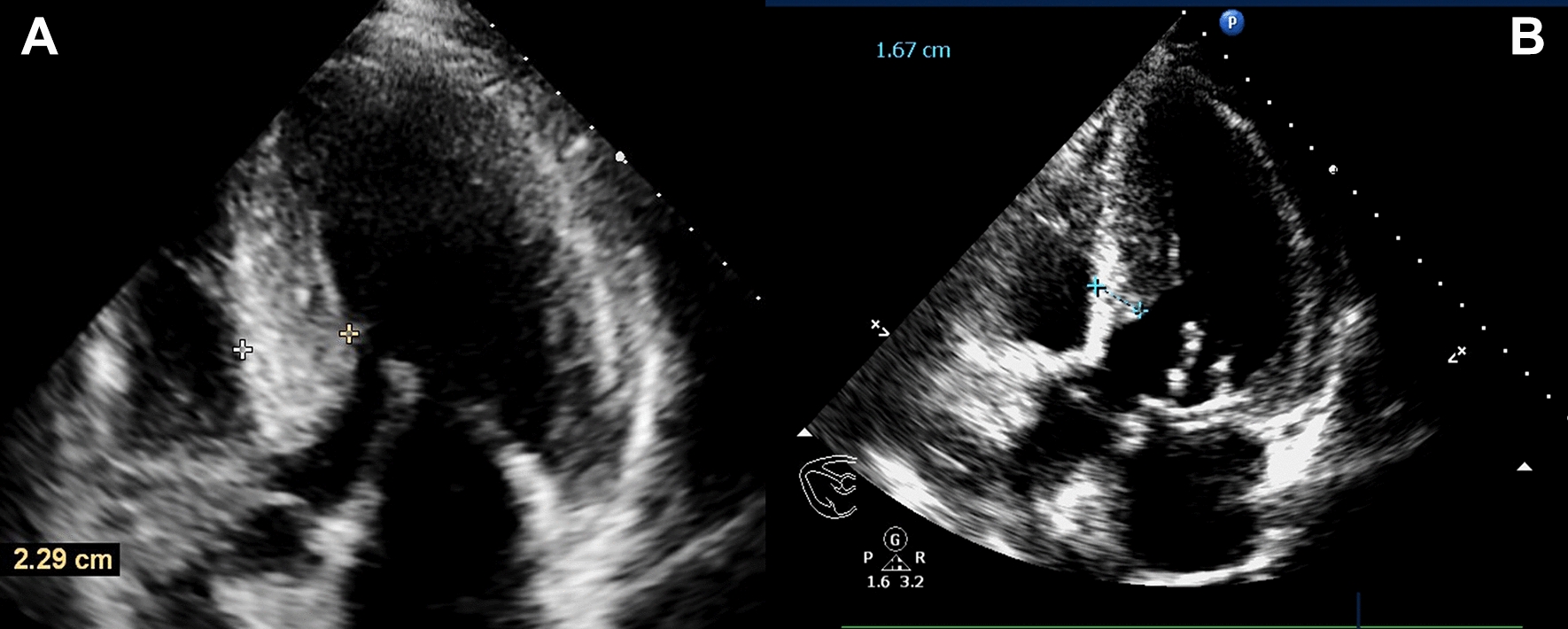
**A** The preoperative septal thickness. **B** The postoperative septal thickness

Male patient, 50-year-old, admitted with the clinical symptoms of persistent syncope and dyspnea and was diagnosed as HOCM. Through regular medical intervention by cardiotonic and diuretic agents and after comprehensive evaluation, the patient was indicated for septal myectomy with the aim of releasing the severe obstruction of LVOT. Preoperatively, both heart function and structural abnormalities were assessed using transthoracic echocardiography (TEE), and based on the findings under which, the asymmetric hypertrophy of interventricular septum and severe mitral regurgitation (MR) complicated with systolic anterior motion of mitral leaflet (SAM) were confirmed. The measured peak pressure difference, maximum flow rate and septal thickness of LVOT were 137 mmHg, 5.84 m/s and 22.9 mm, respectively (Fig. 1). A patient-originated heart printed model using 3D technology (ratio of 1:1) was created in accordance with the exact referred data by CT angiography (Stratasysy J850).

### Assisted Da Vinci robotic surgery

**Fig. 2:**
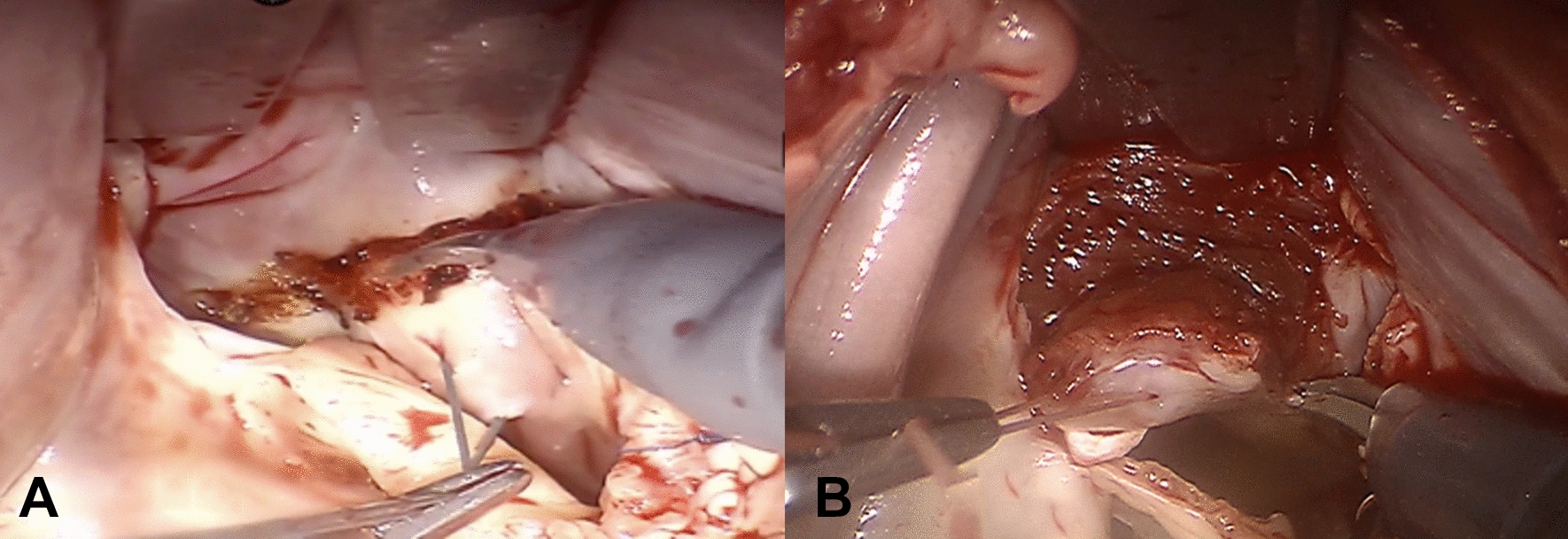
**A** Preset removal hypertrophic lesion. **B** Resection of hypertrophic lesion

**Fig. 3:**
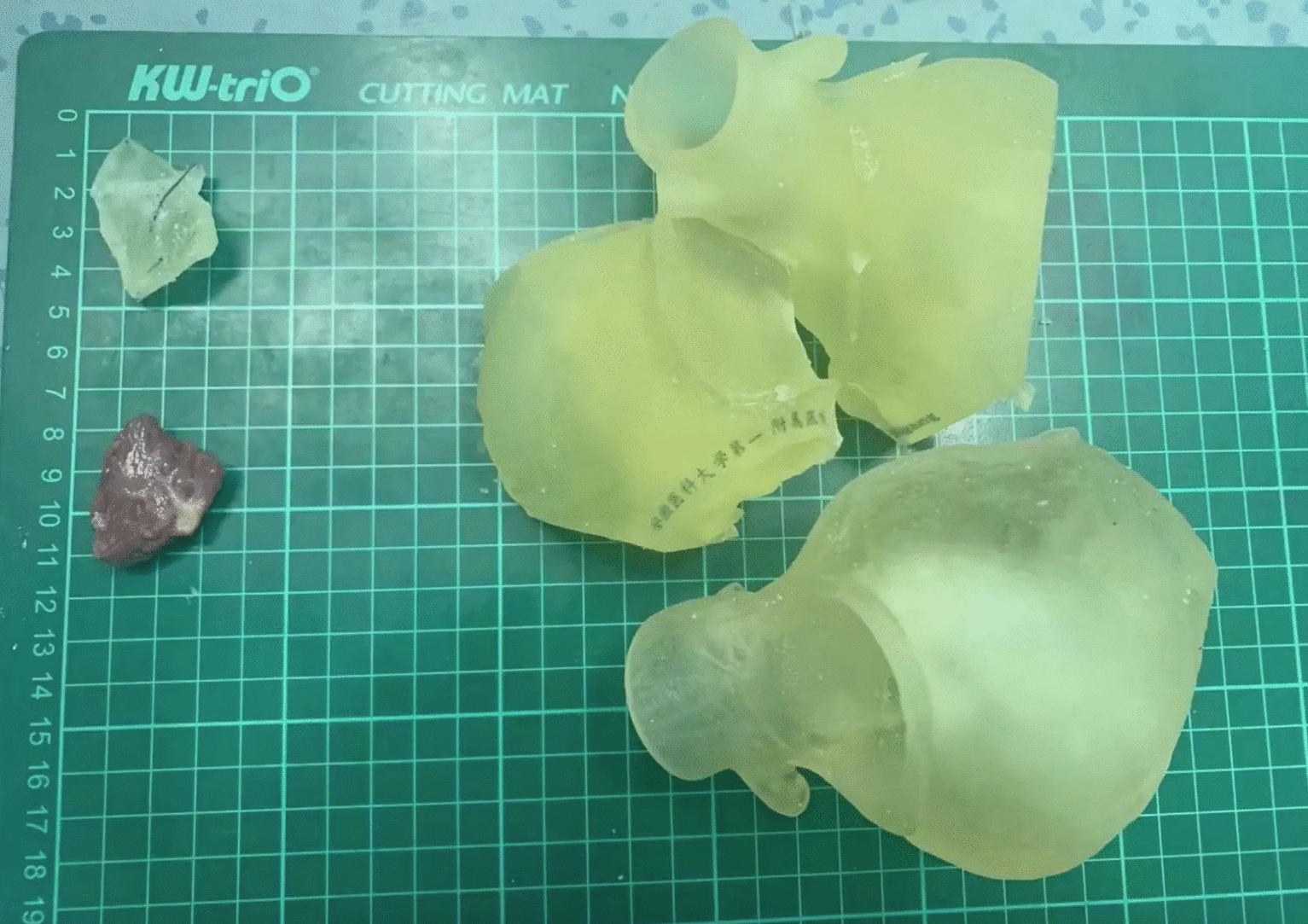
Comparison between virtual and actual resection lesions

The patient was fixed as supine position with relative higher right side padded. The left one-lung ventilation was performed simultaneously. There were three ports with 0.8 cm holed at the 3rd and 6th intercostal space of right anterior line as left-hand, right-hand manipulating sites and the 4th intercostal space of right midclavicular line as atrial retractor site, respectively. All these ports were connected with Trocars which were ready for insertion of mechanical arms. There was one port with 3 cm holed at the 4th intercostal space of right anterior line as working port which was ready for insertion of camera. Peripheral cardiopulmonary bypass (CPB) was established via artery−venous cannulation from femoral artery, vein as well as right internal jugular. After activation of CPB, the mechanical ventilation was ceased to expose the surgical field completely. Carbon dioxide was blew continuously to fulfill the right thoracic cavity via working port. The ascending aorta was clamped by Chitwood and regular perfusion with cardioplegia of Del Nido was processed when temperature < 35 ℃. The left atrium was opened through the incision of groove and suspended by retractor. It was observed that chordae tendineae locating at P3 of posterior leaflet was ruptured and prolapsed. The margin of prolapsed leaflet was sutured with 8-character-pattern suturing. Scattered distribution of white debris was found around the hypertrophic interventricular septum through the incision of anterior mitral leaflet. The depth, thickness and size of hypertrophic myocardium had been preset in accordance with referred information obtained from the individual 3D model. During the surgery, the preset hypertrophic lesion was resected completely and white debris were washed repeatedly (Fig. 2A, B). Importantly, the actual basal thickness was measured again by surgical scissors with the length of 1.0 cm although preoperative assessed value by TEE provided. The extended resection of aortic subvalvular apparatus was about 3 cm with the depth of 1 cm and width of 2.5 cm (Fig. 3). A bovine pericardial patch was used as the substitute of anterior mitral leaflet and sutured at the anterior annulus. Sequentially, a 32^#^ C ring was sutured at the posterior annulus. After mitral valvuloplasty finished, the regurgitation test was negative which indicated that the valve repairing was qualified. Regular rewarming was started and continued until the aorta was opened when temperature > 35 ℃. It was found by TEE after weaning of CPB that mild regurgitation of mitral valve without SAM presented and pressure difference of LVOT was 23 mmHg. Final chest closure and hemostasis were performed after neutralization with protamine.

### Outcome

Postoperatively, based on the findings of echocardiography, both regurgitation and SAM were not found. The measured peak pressure difference, maximum flow rate and septal thickness of LVOT were 15.8 mmHg, 1.99 m/s and 16.7 mm, respectively (Fig. 1B). Under the reasonable postoperative management, the patient recovered smoothly and discharged without any discomforts.

## Discussion

Traditionally, septal myectomy is recommended as the preferred surgical option for releasing left ventricular outflow tract (LVOT) obstruction once the conservative treatment is ineffective or the obstruction-related symptoms recur frequently [2]. Median sternal incision is required as indicated when the standard transaortic approach is processed. However, some particularly anatomical alterations and lesions, especially within the limited surgical field, are not indicated for the traditional incision including smaller aortic annulus, severe septal hypertrophy and prolonged anterior mitral valve, which may lead to, through median sternal incision, inadequate resection, SAM and even damaging of aortic valve [3, 4]. Although some modifications in minimal invasive surgery system including endoscopic and robotic surgery have been applied as the surgical attempts for septal myectomy, due to actual diversities of clinical interventions, the outcomes of patients are highly depended on the surgical ability and experience of surgeons [5, 6].

Recently, 3D printing technology, as an emerging industry, has been the favored and preferred medical mode free from the traditional medical system. Currently, compared with cardiovascular surgery, 3D printing technology has been used widely in thoracoscopic surgery [7–9]. Previously, few literature on the effect and outcome of assisted Da Vinci robotic surgery combined with 3D printing technology in surgical treatment of HOCM has been published. So far as we know, this is the first systematic case presentation of assisted Da Vinci robotic surgery combined with 3D printing technology on surgical treatment for HOCM. In our report, it is promised and potential that accurate and objective evaluation of HOCM before surgery through the created individual 3D model providing varied parameters of heart structure is more suitable and optimal for both surgical improvements during surgery and outcome after surgery. Furthermore, assisted Da Vinci robotic surgery is also a brilliant surgical platform of minimal invasion with more stable operating system, less traumatic injury and faster rehabilitation. Nevertheless, it should be noted that, given the complicated disease of HCOM and the strict requirements of combination surgical strategy, longer learning curve and more mature surgical skill as well as experience are needed.

## Conclusion

The combination strategy of assisted Da Vinci robotic surgery and 3D printing technology is a relative effective and safe surgical option for patients with HOCM with the various advancements including accurate pre-evaluation, procedural stability, complete resection of lesion and minimal invasion.

## Data Availability

The original contributions presented in the study are included in the article/supplementary material, further inquiries can be directed to the corresponding author.
